# Impact of a Web-Based Clinical Decision Support System to Assist Practitioners in Addressing Physical Activity and/or Healthy Eating for Smoking Cessation Treatment: Protocol for a Hybrid Type I Randomized Controlled Trial

**DOI:** 10.2196/19157

**Published:** 2020-09-29

**Authors:** Nadia Minian, Mathangee Lingam, Rahim Moineddin, Kevin E Thorpe, Scott Veldhuizen, Rosa Dragonetti, Laurie Zawertailo, Valerie H Taylor, Margaret Hahn, Wayne K deRuiter, Osnat Melamed, Peter Selby

**Affiliations:** 1 Nicotine Dependence Services Centre for Addiction and Mental Health Toronto, ON Canada; 2 Campbell Family Mental Health Research Institute Centre for Addiction and Mental Health Toronto, ON Canada; 3 Department of Family and Community Medicine University of Toronto Toronto, ON Canada; 4 Dalla Lana School of Public Health University of Toronto Toronto, ON Canada; 5 Applied Health Research Centre Li Ka Shing Knowledge Institute St Michael's Hospital Toronto, ON Canada; 6 Department of Pharmacology and Toxicology University of Toronto Toronto, ON Canada; 7 Department of Psychiatry University of Calgary Calgary, AB Canada; 8 Schizophrenia Division Centre for Addiction and Mental Health Toronto, ON Canada; 9 Institute of Medical Sciences University of Toronto Toronto, ON Canada; 10 Department of Psychiatry University of Toronto Toronto, ON Canada; 11 Banting and Best Diabetes Centre University of Toronto Toronto, ON Canada

**Keywords:** smoking cessation, physical activity, healthy eating, clinical decision support system, hybrid type 1

## Abstract

**Background:**

Modifiable risk factors such as tobacco use, physical inactivity, and poor diet account for a significant proportion of the preventable deaths in Canada. These factors are also known to cluster together, thereby compounding the risks of morbidity and mortality. Given this association, smoking cessation programs appear to be well-suited for integration of health promotion activities for other modifiable risk factors. The Smoking Treatment for Ontario Patients (STOP) program is a province-wide smoking cessation program that currently encourages practitioners to deliver Screening, Brief Intervention, and Referral to treatment for patients who are experiencing depressive symptoms or consume excessive amounts of alcohol via a web-enabled clinical decision support system. However, there is no available clinical decision support system for physical inactivity and poor diet, which are among the leading modifiable risk factors for chronic diseases.

**Objective:**

The aim of this study is to assess whether adding a computerized/web-enabled clinical decision support system for physical activity and diet to a smoking cessation program affects smoking cessation outcomes.

**Methods:**

This study is designed as a hybrid type 1 effectiveness/implementation randomized controlled trial to evaluate a web-enabled clinical decision support system for supporting practitioners in addressing patients’ physical activity and diet as part of smoking cessation treatment in a primary care setting. This design was chosen as it allows for simultaneous testing of the intervention, its delivery in target settings, and the potential for implementation in real-world situations. Intervention effectiveness will be measured using a two-arm randomized controlled trial. Health care practitioners will be unblinded to their patients’ treatment allocation; however, patients will be blinded to whether their practitioner receives the clinical decision support system for physical activity and/or fruit/vegetable consumption. The evaluation of implementation will be guided by the Reach, Effectiveness, Adoption, Implementation, and Maintenance (RE-AIM) framework.

**Results:**

Recruitment for the primary outcome of this study is ongoing and will be completed in November 2020. Results will be reported in March 2021.

**Conclusions:**

The findings of the study will provide much needed insight into whether adding a computerized/web-enabled clinical decision support system for physical activity and diet to a smoking cessation program affects smoking cessation outcome. Furthermore, the implementation evaluation would provide insight into the feasibility of online-based interventions for physical activity and diet in a smoking cessation program. Addressing these risk factors simultaneously could have significant positive effects on chronic disease and cancer prevention.

**Trial Registration:**

ClinicalTrials.gov NCT04223336; https://clinicaltrials.gov/ct2/show/NCT04223336

**International Registered Report Identifier (IRRID):**

DERR1-10.2196/19157

## Introduction

### Background

Modifiable risk factors such as tobacco use, physical inactivity, and poor nutrition account for a significant proportion of the preventable deaths in Canada [1]. These risk factors also frequently cluster together, thereby compounding the risks of morbidity and mortality [2-5]. Up to 93% of Canadians report consistently engaging in two or more unhealthy behaviors [6-8]. Among these risk factors, tobacco use continues to be the single most important modifiable risk factor for health care practitioners to intervene with their patients, as it is associated with the largest reductions in life expectancy and health-adjusted life expectancy [9,10]. Compared to nontobacco users, people who use tobacco are more likely to drink excessive amounts of alcohol, eat fewer fruits and vegetables, and report higher levels of physical inactivity [11,12]. The clustering of these modifiable risk behaviors not only puts individuals at an increased risk for cardiovascular disease and other chronic diseases such as cancer and diabetes but also can have significant impacts on the likelihood of successful smoking cessation [13-18]. In particular, physical activity has been shown to help support smoking cessation by reducing acute cravings and withdrawal symptoms [19-23]. The link between fruit/vegetable intake and smoking cessation is less clear; however, some studies have shown that postcessation weight gain (or the fear thereof) can be a significant barrier to quitting smoking [24-26].

Given this association, smoking cessation programs appear to be well-suited for integration of health-promotion activities for other modifiable risk factors. There are established evidence-based methods for identifying and addressing modifiable risk factors as part of a clinical appointment [27-30]. Specifically, the Screening, Brief Intervention, Referral to Treatment (SBIRT) method has empirical support and requires little time from health care practitioners to deliver (approximately 5-10 minutes) [31]. Moreover, brief interventions have been found to be effective in addressing different types of modifiable risk factors, including physical activity [32] and diet [33]. With respect to the type of behavioral intervention offered, evidence shows that risk communication and encouraging self-monitoring are more effective than other behavior change techniques (eg, provision of educational materials and facilitation) in promoting successful behavior change [34]. However, the impact of implementing SBIRT for other modifiable risk factors within a smoking cessation program is unknown. Since tobacco use is the most important modifiable risk factor for health care practitioners to address, it is important to ensure that interventions for these other risk factors do not reduce the likelihood of quitting smoking successfully.

The Smoking Treatment for Ontario Patients (STOP) program is a province-wide smoking cessation program that provides behavioral counseling and nicotine replacement therapy (NRT) at no cost to the participants. The STOP program has partnered with 153 (83%) family health teams, 61 (81%) community health centers, and 18 (75%) nurse practitioner-led clinics in Ontario. These primary care settings are not paid to offer the program but are provided with no-cost NRT to dispense to participants as needed. Treatment is tailored by the health care practitioner and patients are eligible for up to 26 weeks of NRT during their time in the program (12 months). Health care practitioners will typically meet with participants every 2-4 weeks to assess their progress and up to 4 weeks of NRT is dispensed in any given visit. The behavioral counseling provided by the health care practitioners is primarily guided by the principles of motivational interviewing [35]. In addition, STOP health care practitioners are encouraged to deliver SBIRT to patients who are experiencing depressive symptoms [36] or consuming excessive amounts of alcohol [37] via a web-enabled clinical decision support system (CDSS). However, there is no built-in CDSS for physical inactivity and poor diet.

A CDSS is a computer app designed to present patient-specific, actionable information to help support practitioners in determining diagnoses and treatment approaches [38,39]. Given the increased use of electronic medical records in primary care offices, broader implementation of CDSSs has become possible, offering an opportunity to improve evidence-based care.

### Objectives

The aims of this study are to: (1) assess whether adding a CDSS for physical activity and diet to a smoking cessation program affects smoking cessation outcomes, and (2) quantitatively and qualitatively assess the implementation of the study using the Reach, Effectiveness, Adoption, Implementation, and Maintenance (RE-AIM) framework.

## Methods

### Trial Design

The study will utilize an effectiveness/implementation hybrid type 1 design for the evaluation [40]. This design was chosen as it allows for simultaneous testing of the intervention, its delivery in target settings, and the potential for implementation in real-world situations; using a setting in which there is already clinical momentum reduces risks with potentially high benefit [40]. This trial will be made operational via the STOP program. Intervention effectiveness will be measured using a two-arm randomized controlled trial. Health care practitioners seeing patients randomized to the intervention arm (Group A) will receive computer alerts when a patient does not meet the national guidelines of nutrition or physical activity. They will then be guided to deliver a brief intervention to the patient. Health care practitioners seeing patients randomized to the treatment-as-usual arm (Group B) will not receive computer alerts for physical activity and fruit/vegetable consumption. The evaluation of implementation will be guided by the RE-AIM framework [41].

### Preimplementation

The implementation of this project will be guided by the Interactive Systems Framework (ISF) for dissemination and implementation. The ISF suggests that there are three interacting systems involved in translating research findings to practice: synthesis and translation systems, support systems, and delivery systems [42]. In this study, the delivery systems will be the health care practitioners in primary care clinics, and people who smoke and who do not meet the national guidelines of diet or physical activity will be the recipients of the intervention.

At the time of submission of this manuscript, we had already conducted two community-informed engagement events with the target population (STOP program participants) to cocreate risk communication messages that health care practitioners could use with their patients and a self-monitoring resource for patients to track their risk behaviors. These resources are based on best practices and patient experiences, and form part of the ISF synthesis and translation systems [42]. As part of the support system, we developed and delivered an interactive webinar training for health care practitioners that combined the latest evidence on behavior change approaches and the findings from the engagement events with STOP participants. The webinar provides concrete advice as to why and how health care practitioners should address physical activity and fruit/vegetable consumption, and how these aspects can be incorporated in a smoking cessation treatment program. In the past, STOP health care practitioners have expressed reservations toward addressing multiple behaviors; therefore, this webinar also trains health care practitioners on how to address 2-3 behaviors simultaneously to produce maximum change in participants [43].

### Participants

Participants will be treatment-seeking smokers that enroll in the STOP program. Individuals interested in enrolling in the program either self-refer or are referred by their health care practitioner. Clinics enroll their patients into the STOP program using the STOP portal, an online portal that allows for data collection and management. The current portal already has a CDSS [44,45] to guide health care practitioners with the delivery of a brief intervention for patients who also have current or past depression, as defined by the Patient Health Questionnaire (PHQ-9) [46], or consume excessive amounts of alcohol, as defined by the Alcohol Use Disorders Identification Test (AUDIT-C and AUDIT-10) [47].

At the time of enrollment, all patients are asked to provide informed consent prior to seeing their health care practitioner. Using the online portal, the health care practitioner completes the baseline enrollment survey with the patient. This survey has screening questions for tobacco use and related measures, including alcohol use [47], depression [46], physical activity [48], and fruit/vegetable consumption [49].

The implementers of this CDSS pathway will include health care practitioners from a wide variety of disciplines (eg, nurses, pharmacists, social workers) who have been trained by the STOP team. They also belong to the STOP program’s Community of Practice where they engage in continuous learning through informal (eg, listserv) and formal (eg, mentoring phone calls, webinars) mechanisms.

### Setting and Location

The trial will take place in family health teams (n=153), community health centers (n=61), and nurse practitioner–led clinics (n=18) in Ontario, Canada implementing the STOP program and using STOP’s online portal as of November 29, 2019.

### Inclusion Criteria

When a patient is enrolling in the STOP program, the STOP enrollment survey must be delivered by the health care practitioner using the online STOP portal. At the time of enrolling in the STOP program, patients must also be below the national guidelines for physical activity [48] or fruit/vegetable consumption [49]. A low level of physical activity is defined as engaging in less than 150 minutes of moderate/vigorous exercise per week [48]. A low level of fruit/vegetable consumption is defined as consuming less than 7 servings (women) or 8 servings (men) of fruit/vegetables daily in accordance with the 2007 Canadian Food Guide [49].

Patients must also provide at least one piece of contact information (phone number or email address) to allow for follow up to be conducted at 6 months.

### Randomization

Patients will be randomly allocated at the time of enrollment to the intervention arm (Group A) or control arm (Group B) at a 1:1 allocation ratio. The random allocation sequence is generated automatically and internally by the STOP portal. The study team, health care practitioners, and participants do not have access to this automatically generated randomization. Health practitioners treating participants randomized to Group A will have a CDSS for physical activity and fruit/vegetable consumption available to them, whereas participants randomized to Group B will receive treatment as usual in the STOP program.

### Blinding

Health care practitioners will not be blinded to the patient’s treatment allocation, and they will see both intervention and control patients. Patients will be blinded to whether their health care practitioner receives the CDSS for physical activity and/or fruit/vegetable consumption.

### Intervention

As described above, the STOP portal currently has a CDSS that screens patients and alerts health care practitioners when their patients report risky alcohol use [44] or depressive symptoms [45]. Health care practitioners are then prompted to address these risk factors as part of the patient’s smoking cessation treatment. Both Group A (intervention) and Group B (control) will continue to have the CDSS for alcohol use and depressive symptoms.

#### Intervention (Group A)

**Figure 1:**
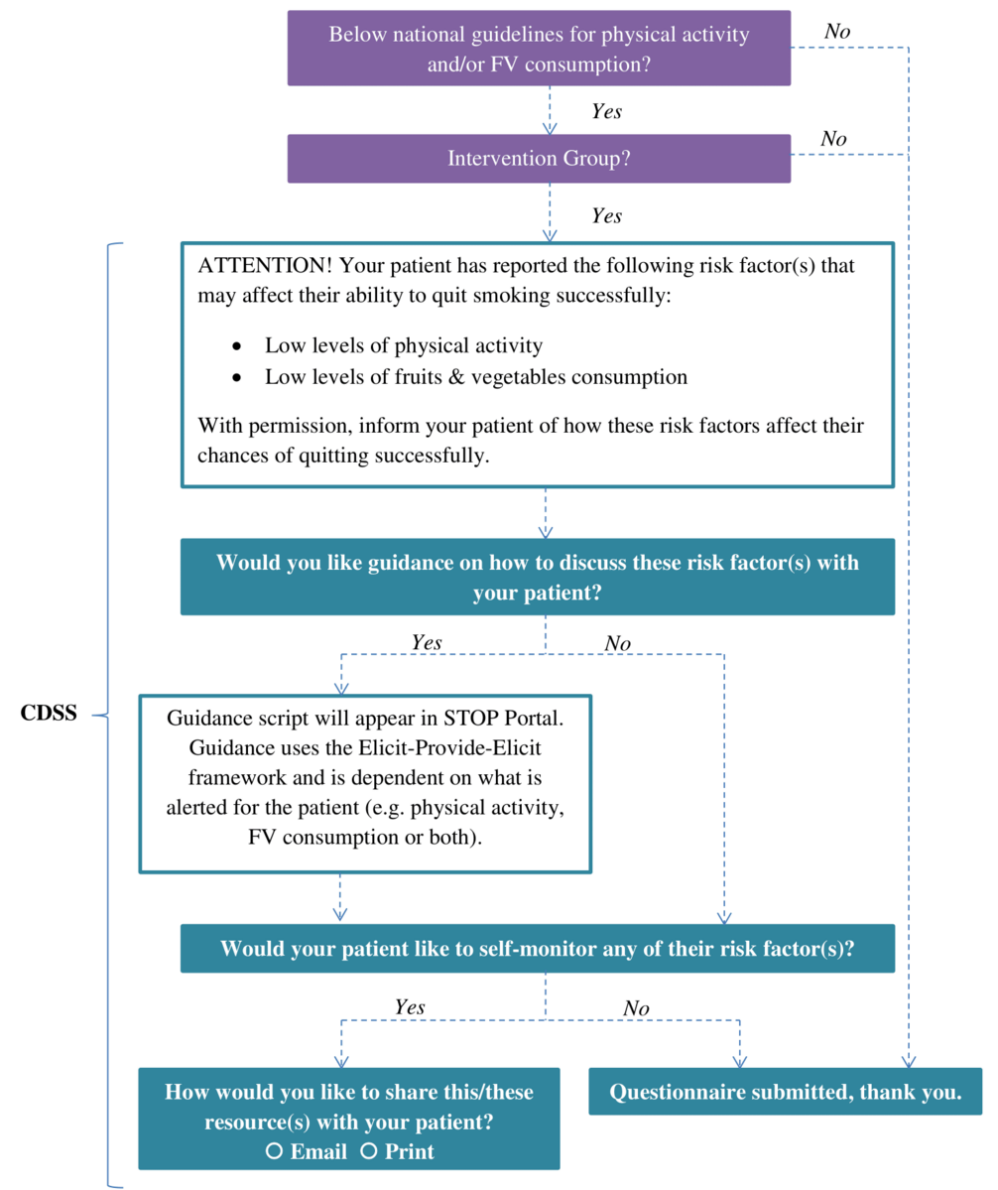
Study workflow diagram. FV: fruits and vegetables.

The STOP portal will be adapted to provide a CDSS for physical activity and/or fruit/vegetable consumption (Figure 1). At the time of enrollment, the CDSS will: (1) screen patients for their physical activity and fruit/vegetable consumption levels, (2) provide health care practitioners with an alert when the patient is not meeting guidelines for physical activity and fruit/vegetable consumption, and (3) guide health care practitioners to intervene with the patient using the following steps.

Step 1 involves risk communication with their patient to raise awareness of the identified risk factors. This includes sharing the information in the alert, which lists the specific risk factors (eg, low levels of physical activity) for the patient, and discussing how each risk factor affects their patient’s health and smoking cessation treatment. The CDSS also includes optional guidance for health care practitioners on how to provide risk communication using the Elicit-Provide-Elicit framework [50]. The guidance message will remind health care practitioners to use positively framed risk communication messages. This was based on the results of the engagement events with STOP participants.

In step 2, a customized self-monitoring resource for the risk factors is provided to patients who want to self-monitor as a part of their smoking cessation treatment. The self-monitoring resource (see Multimedia Appendix 1), which health care practitioners will be able to print or email to their patients, is a 1-page weekly tracking sheet, including the following key features: ability to track multiple behaviors simultaneously on the same page; ability to record the number of cigarettes smoked, in addition to the other behaviors they chose to monitor; and setting weekly goals.

#### Control (Group B)

Health care practitioners of patients randomized to Group B will experience the STOP portal as usual. STOP participants will also be asked screening questions for physical activity and diet; however, health care practitioners are not provided with an alert about their physical activity and fruit/vegetable consumption levels, and are not prompted to take any actions to address these two behaviors. Practitioners treating patients in Group B are only provided with the CDSS for depressive symptoms and alcohol use, which has been available in the STOP portal prior to this trial.

### Outcomes

The primary outcome of the study is self-reported smoking cessation at 6-month follow up. This dichotomous outcome will be measured by a negative response to the 7-day point prevalence abstinence question: “Have you smoked a cigarette, even a puff, in the last 7 days.” Researchers have shown that self-reporting for smoking status is highly consistent with biochemical assessments of smoking [51-54].

The secondary outcomes of the study will be a self-reported change in physical activity levels and fruit/vegetable consumption levels from baseline to 6-month follow up. Changes in physical activity will be assessed using an adapted Exercise Vital Signs screener [55] that consists of two questions: “On average, how many days per week do you engage in moderate-to-strenuous (vigorous) exercise (like a brisk walk)?” and “On these days, for how many minutes do you typically exercise at this level?” This screener has been validated against step counts [55]. Changes in fruit and vegetable consumption will be measured using a single question: “In a typical day, how many total servings of fruits and vegetables do you eat? (1 serving is 1/2 cup of fresh, frozen, or canned fruits or vegetables, or 1/2 cup of 100% juice. Please DO NOT include potatoes).”

These questions will be administered at the time of enrollment in the STOP program and at the 6-month follow up. Responses to the self-report questions for both the primary and secondary outcomes will be collected via phone or email. We will use the RE-AIM framework to organize our implementation evaluation, which is summarized in Textbox 1.

**RE-AIM framework for implementation and evaluation of the trial.**

- REACH:
  Has the reach of the STOP portal changed? (The reach will be defined as the number of STOP enrollments completed online).
- EFFECTIVENESS:
  What proportion of participants from the intervention vs control groups report smoking cessation at 6-month follow up? (please see Outcomes section for details)
- ADOPTION:
  What proportion of intervention group participants received risk communication and at least one self-monitoring resource?
- IMPLEMENTATION:
  What proportion of participants in Group A accepted the self-monitoring resource for physical activity and/or fruits/vegetables consumption?
  What are the barriers and facilitators health care practitioners report to delivering the intervention as intended?
  What are some barriers and facilitators faced by participants in receiving the intervention?
- MAINTENANCE:
  What proportion of intervention participants who were quit at 6 months were also quit at the 12-month follow up?

### Sample Size

A sample size of 3998 (1999 per group) is needed to detect a clinically meaningful difference in smoking cessation outcome between groups at 6 months. Using past STOP data, and after accounting for nonresponse, we estimate the proportion of patients who will quit smoking at 6 months to be 0.26, and set α to .05 and the power to 80%. For the effect size, we use an absolute difference in proportions of 0.04, which is an established standard for clinical significance in smoking cessation [56].

Based on past experience in conducting trials using the STOP platform, we anticipate a loss to follow up of 20%. This increases the necessary total baseline sample to 4998 (2499 per group). Based on enrollment in 2017-2018, we estimate that the required sample size for the primary outcome will be achieved in approximately 7 months. However, as the recruitment and follow-up periods are likely to overlap slightly, we will monitor the follow-up rate being achieved. If this rate is lower than anticipated, we will consider extending recruitment to achieve an adequately powered analysis sample.

### Statistical Analysis

Analysis of the primary and secondary outcomes will be performed using the intention-to-treat principle. Patients will be analyzed in the study arm to which they are assigned (control vs intervention group). Descriptive statistics (without testing) will be used to summarize patient-level baseline characteristics in the intervention and control groups.

We will test for a group difference in our primary outcome using a chi-square test. For our secondary outcomes, we will test for group differences in change over time by regressing the 6-month outcomes on the baseline measures and group. We will use linear regression for total exercise time and ordinal logistic regression for total fruit and vegetable consumption.

Although the STOP program comprises many separate clinical sites, analyses using mixed-effects models indicate that any true site effects are quite small. We therefore do not anticipate a need to account explicitly for clustering. However, we will test this by measuring the site-level intraclass correlation, and will analyze our data using random intercept models if deemed to be appropriate.

To understand wider associations between our outcomes and participant characteristics, we will also perform multivariate analyses, including logistic regression for smoking cessation, linear regression for exercise, and ordinal logistic regression for fruit and vegetable consumption. In these models, we will include education, income, substance use, mental health diagnoses, gender, age, and the Heaviness of Smoking Index [57] as independent variables.

Primary and secondary outcomes will only be available from patients who complete a 6-month follow-up survey. As a result, we anticipate that approximately 20% of these will be missing, and that there will also be some missing values in baseline variables. We will address these missing data using multiple imputation with chained equations. Our missing data model will include all 3 outcomes, the variables from our secondary (adjusted) analyses, and auxiliary variables, including smoking status from STOP follow-up assessments performed at 3 and 12 months after baseline (where available), smoking status at the last clinical visit, number of visits, clinical counseling received, and type and duration of NRT prescribed. A statistical analysis plan will specify the number of imputed datasets to be created, the models to be used, and the variables to be incorporated.

### Data Security and Quality Assurance

Data will be stored in secure computerized files and the informed consent forms will be kept in locked cabinets in the study team’s locked offices. The STOP portal uses two different database encryption mechanisms (transparent database encryption and cell-level encryption) to protect the online data from intrusion. The study team will conduct monthly review of the data on the STOP portal to ensure the CDSS continues to operate correctly throughout the study.

### Ethical Approval and Trial Status

This study was reviewed by the Research Ethics Board at the Centre for Addiction and Mental Health, Toronto, Ontario, Canada (119-2018). This trial is registered with ClinicalTrials.gov (NCT04223336).

## Results

As of April 2020, interactive webinar training was delivered to STOP health care practitioners, and recruitment has started but is not yet complete. We estimate that recruitment will be completed in November 2020. Results will be reported in March 2021.

## Discussion

This clinical trial provides a novel method for addressing physical activity and fruit/vegetable consumption as part of a smoking cessation program. Smoking cessation programs continue to be essential public health initiatives. The literature shows that with every two individuals who quit smoking, at least one life is saved from a tobacco-related death [56]. Furthermore, in Ontario, physical inactivity and inadequate diet contribute to approximately 40% of all deaths [10]. If these risk factors can be addressed simultaneously, it could have significant positive effects on public health.

Moreover, delivery of this intervention via a web-based portal allows for rapid, system-wide implementation. The STOP program enrolls over 23,000 participants per year and the impact of this intervention could be widespread. The findings from this study can help inform future development and integration of interventions for other modifiable risk factors within smoking cessation programs.

We chose to conduct a hybrid type I design since our primary aim is to test the effects of a clinical intervention (adding a CDSS for physical activity and fruit/vegetable consumption) on relevant outcomes (smoking cessation at 6 months, and change in physical activity levels and fruit/vegetable consumption levels from baseline to 6 months). However, we also want to gather information on implementation outcomes from the study, including whether it changes the reach of the program, the adoption of the intervention (whether health care practitioners follow the recommendations of the CDSS and patients accept the resource), and potential barriers and facilitators to real-world implementation of the intervention. This design will provide high reward as the implementation research offers additional context that will help with understanding whether a clinical trial is effective or ineffective.

We chose to evaluate effectiveness via a randomized controlled trial with randomization at the individual level, as this is feasible (in both arms, the health care practitioner can respond directly based on the CDSS) and provides more power compared to cluster randomized controlled trials for detecting any clinically significant changes in the outcomes of interest [58].

There are a few potential study limitations that need to be acknowledged. Health care practitioners will not be blinded to the patient’s treatment allocation and will be seeing both intervention and control patients. The lack of blinding can lead to possible group contamination as health care practitioners may apply their knowledge from the intervention to the control group. The potential learning effect may decrease the effect of the trial. However, the self-monitoring resource for physical activity and diet are only accessible to intervention patients, which will help to reduce the cross-contamination. Randomization at the organizational level would not completely eliminate the risk of contamination as some health care practitioners work at multiple organizations.

The results of this trial will be disseminated through peer-reviewed publications and conference presentations. Results will also be presented and communicated appropriately to STOP health care practitioners and the funders for this initiative.

The findings of the study will provide much needed insight into whether other modifiable risk factors can be addressed simultaneously as a part of a smoking cessation program without affecting the individual’s ability and success in quitting smoking. If the results of the study are positive (ie, more intervention group individuals quit smoking) or show no negative effect on smoking cessation, we can continue to offer physical activity and diet interventions as part of smoking cessation programs. Our evaluation of the implementation of the study will also provide insights into whether CDSS interventions are an appropriate method for promoting physical activity and diet interventions as part of smoking cessation treatment.

## Appendix

Multimedia Appendix 1 Self-monitoring resource.

Multimedia Appendix 2 CONSORT E-HEALTH checklist (v 1.6.1).

## Abbreviations

AUDIT: Alcohol Use Disorders Identification Test
CDSS: Clinical Decision Support System
ISF: Interactive Systems Framework
NRT: nicotine replacement therapy
RE-AIM: Reach, Effectiveness, Adoption, Implementation, and Maintenance
SBIRT: Screening, Brief Intervention, Referral to Treatment
STOP: Smoking Treatment for Ontario Patients
