# Supplementary material for: Impact of a Web-Based Clinical Decision Support System to Assist Practitioners in Addressing Physical Activity and/or Healthy Eating for Smoking Cessation Treatment: Protocol for a Hybrid Type I Randomized Controlled Trial
Source: JMIR Res Protoc. 2020 Sep 29;9(9):e19157. doi: 10.2196/19157 (PMC7556369; doi:10.2196/19157)
Supplement: Multimedia Appendix 1 [file resprot_v9i9e19157_app1.pdf]

APPENDIX 1

My Tracking Sheet

Smoking								
**Goal: No more than _____ cigarettes per day and _____ per week.**								
**Week starting:**	**Sunday**	**Monday**	**Tuesday**	**Wednesday**	**Thursday**	**Friday**	**Saturday**	**Total:**
mm/dd: ______								
**Comments:**								

Physical Activity								
**Goal: I plan to do moderate-to-vigorous exercise for _____ min _____ times or more each week.** *Examples of moderate-to-vigorous exercise include brisk walking, bike riding, jogging.*								
**Week starting:**	**Sunday**	**Monday**	**Tuesday**	**Wednesday**	**Thursday**	**Friday**	**Saturday**	**Total:**
mm/dd: ______								
**Comments:**								

Fruits and Vegetables								
**Goal: I plan to eat _____ servings of fruits and vegetables per day** *1 serving is 1/2 cup of fresh, frozen, or canned fruits or vegetables. Please DO NOT include potatoes.*								
**Week starting:**	**Sunday**	**Monday**	**Tuesday**	**Wednesday**	**Thursday**	**Friday**	**Saturday**	**Total:**
mm/dd: ______								
**Comments:**								

Development of this tracking sheet was funded by the Public Health Agency of Canada and the Medical Psychiatry Alliance.
